# Safety evaluation of Tofersen in amyotrophic lateral sclerosis based on the FAERS database

**DOI:** 10.3389/fneur.2026.1843630

**Published:** 2026-05-22

**Authors:** Xuefeng Xiao, Gang Hao, Ping Wan, Wenzhuang Hou

**Affiliations:** 1 Suzhou Polytechnic University, Suzhou, Jiangsu, China; 2 Affiliated Mental Health Center of Jiangnan University, Wuxi, Jiangsu, China; 3 Affiliated Huishan Hospital of Xinglin College, Nantong University, Wuxi Huishan District People’s Hospital, Wuxi, Jiangsu, China

**Keywords:** adverse events, amyotrophic lateral sclerosis, data mining, FAERS, Tofersen

## Abstract

**Background:**

This study utilizes data from the US Food and Drug Administration Adverse Event Reporting System (FAERS) to conduct a post-marketing safety evaluation of Tofersen.

**Methods:**

A systematic search of the FAERS database was performed to identify adverse event (AE) reports from Q1 2023 to Q4 2025 was performed. Multi-dimensional disproportionality analyses were conducted using the ROR, PRR, BCPNN, and MGPS method.

**Results:**

A total of 409 Tofersen-related reports were identified, revealing significant signals across 22 System Organ Class (SOC) categories and 369 Preferred Terms (PTs). At the SOC level, the most prominent safety signals were observed in injury, poisoning and procedural complications, nervous system disorders, and musculoskeletal and connective tissue disorders. In contrast, ear and labyrinth disorders, as well as respiratory, thoracic and mediastinal disorders, were not listed in the current product labeling and may warrant further investigation. At the PT level, neurological procedural complication, CSF red blood cell count positive, CSF white blood cell count increased, and CSF cell count increased exhibited the strongest signals, primarily reflecting cerebrospinal fluid cytological and biochemical abnormalities, as well as procedural-related adverse events such as post-lumbar puncture syndrome, procedural pain, and procedural headache. Although central nervous system-related events (e.g., increased intracranial pressure, peroneal nerve palsy, papilloedema, and facial paralysis) and infectious or inflammatory events (e.g., radiculopathy, myelitis, aspiration pneumonia, and meningitis) were less frequently reported, their relatively high disproportionality signals warrant clinical attention and systematic monitoring. Notably, a newly identified signal of pulmonary embolism suggests a potential thromboembolic risk in specific patient populations.

**Conclusion:**

These findings provide real-world evidence to inform the balance between the therapeutic potential and safety profile of Tofersen. Future clinical strategies should focus on mitigating central nervous system-related and procedure-related adverse events. Further mechanistic studies are crucial.

## Highlights

- This FAERS-based study provides the first comprehensive real-world safety profile of Tofersen.
- It identifies strong signals for CSF abnormalities, neurological complications, and procedural-related adverse events.
- A novel signal of pulmonary embolism is detected, highlighting the need for targeted monitoring.

## 1 Introduction

Amyotrophic lateral sclerosis (ALS) is a highly heterogeneous and fatal neurodegenerative disease characterized by the selective and progressive loss of cortical pyramidal cells, brainstem motor neuron nuclei, spinal anterior horn motor neurons, and corticospinal tracts (1). The disease progresses rapidly, with a median survival time from symptom onset to death of only 36 months (2), and most patients die within 3 to 5 years due to respiratory muscle paralysis and related complications (3). Epidemiological studies indicate that the global age-standardized incidence rate of ALS is 1.68 per 100,000 people (4), with approximately 30,000 deaths annually (5), placing a substantial burden on both patients and society. Although various therapeutic interventions are currently available, ALS still lacks a curative treatment, and existing medications can only partially alleviate symptoms or delay disease progression. Tofersen is a specific treatment for ALS caused by superoxide dismutase 1 (SOD1) gene mutations, which works by reducing the levels of abnormal SOD1 protein and plasma neurofilament light chain (NfL), thereby significantly slowing the progression of neurodegenerative damage (6). In April 2023, Tofersen was approved by the U. S. FDA as the first therapy for ALS caused by SOD1 gene mutations, marking an important breakthrough in the field of ALS treatment.

However, like other drugs, Tofersen may cause a range of potential adverse events (AE), particularly given its intrathecal route of administration. The current safety evidence for Tofersen primarily comes from the phase III clinical trial VALOR (NCT02623699) and its open-label extension (OLE) study. Data from the VALOR study show that the most common AEs in the Tofersen treatment group were neuralgia, fatigue, and headache (7). Notably, serious AEs included inflammatory neurological events such as myelitis and radiculitis (8). The incidence of AEs related to lumbar puncture, such as pain at the puncture site and cerebrospinal fluid leakage, was significantly higher in the Tofersen group (9). It is important to note that the drug has been on the market for a relatively short period, and the current safety data primarily derive from strictly controlled clinical trials. As clinical use expands, potential risks in special populations (such as patients with hepatic or renal dysfunction, or those receiving concomitant medications) may emerge in real-world settings.

It is worth mentioning that in October 2024, Tofersen received breakthrough approval from the National Medical Products Administration (NMPA) in China, becoming a new treatment option for ALS in the region (10). This is undoubtedly good news for patients, but the high price also poses a significant burden on consumers. The cost of one dose of Tofersen is $14,230, and 14 doses are required in the first year, totaling $199,200 (approximately 1.4 million RMB). Furthermore, research and post-marketing safety data on Tofersen in the Chinese population remain relatively scarce (11), and more empirical studies are urgently needed to fill this gap. Fortunately, a multi-center, non-interventional post-marketing surveillance study of Tofersen has already begun (8), providing a foundation for further evaluation of its clinical safety in the Chinese market. As its use expands, more research on the safety of Tofersen will be needed to assess the cost–benefit balance for patients.

Given its unique therapeutic mechanism, potential risks, and high cost, monitoring the long-term AEs of Tofersen is crucial. The US FDA Adverse Event Reporting System (FAERS) is an important voluntary reporting database widely used for monitoring adverse drug reactions and safety signals (12). FAERS features extensive coverage, a large volume of data, and real-time updates, providing essential technical support and abundant real-world evidence for post-marketing drug safety monitoring. This study utilizes the FAERS database to systematically analyze the AEs of Tofersen, helping to identify potential safety issues and providing scientific evidence for rational clinical use and regulatory decision-making.

## 2 Materials and methods

### 2.1 Data source

Considering the timeline of drug introduction to the market, this study extracted all reports containing the search terms “Tofersen” and “Qalsody” from the FAERS database, covering surveillance data from the first quarter of 2023 to the fourth quarter of 2025. This timeframe corresponds to the post-marketing surveillance period following the FDA’s accelerated approval of Tofersen (brand name Qalsody®) in April 2023. The raw data files included DEMO (Demographic Information), DRUG (Drug Information), REAC (Adverse Event Information), OUTC (Outcome Information), RPSR (Report Source Information), THER (Treatment Information), and INDI (Indication Information), all of which were downloaded from the FDA’s official website.

### 2.2 Data extraction and analysis

During the data cleaning process, duplicate reports were removed according to FDA-recommended methods: each CASE_ID was used as a unique identifier, and records with the highest PRIMARY_ID and the most recent report date were retained, ensuring the uniqueness and accuracy of the data. Only reports clearly indicating Tofersen as the “primary suspect (PS)” drug were included. Reports with Preferred Terms (PT) ≥ 3 were selected. The filtered data covered multiple dimensions, including patient demographics (sex, age, weight), suspected drug information, system organ class classification of AEs, event severity, report timing, and geographical distribution. All AE terms were standardized and mapped using the Medical Dictionary for Regulatory Activities (MedDRA) to ensure consistency and standardization (13).

### 2.3 Signal detection methods

To identify potential AE signals related to Tofersen, this study employed four commonly used pharmacovigilance signal detection methods, including Reporting Odds Ratio (ROR), Proportional Reporting Ratio (PRR), Bayesian Confidence Propagation Neural Network (BCPNN), and the Multi-dimensional Gamma-Poisson Shrinkage Algorithm (MGPS). Among these, ROR and PRR, as classic non-Bayesian methods, have high sensitivity in early signal detection; BCPNN effectively reduces data noise by incorporating Bayesian prior distributions, thereby improving signal specificity; MGPS overcomes the limitations of traditional methods in data sparsity and multidimensional signal detection by combining polynomial distribution modeling with Bayesian shrinkage techniques (14). This study combined these four disproportionality analysis methods to complementarily expand signal detection dimensions, enabling a more comprehensive evaluation of AE data. Table 1 presents the detailed formulas and threshold criteria for these methods, along with the 2 × 2 contingency table. The results of each algorithm are positively correlated with AE signal strength, with higher values indicating stronger signals and a greater potential association between the target drug and AEs.

**Table 1:** A two-by-two contingency table and detailed formulas for disproportionality analysis.

Category	Tofersen-related AEs	Other AEs	Sums
Tofersen	a	b	a + b
Other drugs	c	d	c + d
Sums	a + c	b + d	a + b + c + d
Algorithms	Calculation formulas		
ROR	$\mathrm{ROR}=\frac{\mathrm{ad}}{\mathrm{bc}}$		a ≥ 3 and 95% CI (lower limit) > 1
	$95\%\mathrm{CI}=e^{\ln(\mathrm{ROR})\pm 1.96\sqrt{\frac{1}{a}+\frac{1}{b}+\frac{1}{c}+\frac{1}{d}}}$		
PRR	$\mathrm{PRR}=\frac{a(c+d)}{c(a+b)}$		a ≥ 3 and 95% CI (lower limit) > 1
	$95\%\mathrm{CI}=e^{\ln(\mathrm{PRR})\pm 1.96\sqrt{\frac{1}{a}-\frac{1}{a+b}+\frac{1}{c}-\frac{1}{c+d}}}$		
	$\chi^{2}=\frac{\left[{\left(\mathrm{ad}-\mathrm{bc}\right)}^{2}](a+b+c+d\right)}{\left[(a+b)(c+d)(a+c)(b+d)\right]}$		
BCPNN	$\mathrm{IC}=\log_{2}\frac{a(a+b+c+d)}{\left(a+c)(a+b\right)}$		IC025 > 0
	$E(\mathrm{IC})=\log_{2}\frac{\left(a+\gamma 11)(a+b+c+d+\alpha )(a+b+c+d+\beta \right)}{\left(a+b+c+d+\gamma )(a+b+\alpha 1)(a+c+\beta 1\right)}$		
	$V(\mathrm{IC})=\frac{1}{{\left(\ln2\right)}^{2}}\{[\frac{(a+b+c+d)-a+\gamma -\gamma 11}{\left(a+\gamma 11)(1+a+b+c+d+\gamma \right)}]+[\frac{(a+b+c+d)-(a+b)+\alpha -\alpha 1}{\left(a+b+\alpha 1)(1+a+b+c+d+\alpha \right)}]+[\frac{(a+b+c+d)-(a+c)+\beta -\beta 1}{\left(a+c+\beta 1)(1+a+b+c+d+\beta \right)}]\}$		
	$95\%\mathrm{CI}=\mathrm{IC}025=E(\mathrm{IC})-2\sqrt{V(\mathrm{IC})}$		
	$\gamma =\gamma 11\frac{\left(a+b+c+d+\alpha )(a+b+c+d+\beta \right)}{\left(a+b+\alpha 1)(a+c+\beta 1\right)}$		
MGPS	openEBGM package inR		EBGM05 > 2

EBGM and its confidence intervals (EBGM05, EBGM95) were calculated using the openEBGM package (version 0.9.1) in R.

To improve clinical interpretability, PTs related to the underlying indication were identified and handled separately. Indication-related PTs were defined as terms reflecting the natural history, core clinical manifestations, or disease progression of ALS. Given the complexity and heterogeneity of ALS, identification of these PTs was informed by the clinical features outlined in Panel 1 of the review by Eva L. Feldman (15).

Classification was conducted in accordance with these predefined clinical principles during data interpretation. PTs meeting these criteria were considered indication-related and were not subjected to further discussion or analysis. To ensure completeness of the results, these indication-related PTs were marked with a superscript “a” and were not excluded from the results section.

### 2.4 Statistical analysis

Descriptive analysis methods were used to analyze the results. Data storage and structured queries were performed using MySQL 8.0, while data organization and preprocessing were completed using Microsoft Excel 2016. Visualization and statistical analyses were conducted using R (version 4.4.1). Specifically, EBGM, EBGM05, and EBGM95 values were estimated using the MGPS model implemented in the openEBGM package (version 0.9.1).

### 2.5 Ethics approval and consent to participate

The FAERS database is a publicly available and anonymized dataset; therefore, ethical approval and informed consent were not required for this study.

## 3 Results

### 3.1 Basic information on Tofersen-related adverse events

A total of 409 relevant AE reports were collected. Demographic characteristics showed that male patients accounted for 47.4% (194 cases), slightly higher than female patients at 43.0% (176 cases). In terms of age distribution, the 18–65 age group had the highest proportion (43.0%); however, 40.1% of cases had missing age information, which limited further evaluation of the relationship between age and AEs. Geographical distribution analysis showed that the United States reported the most cases, accounting for 72.9%, followed by Italy (5.7%). Regarding report sources, consumer self-reports accounted for 47.4%, with medical professionals (including physicians at 29.3% and other health professional at 11.7%) and pharmacists (4.6%) making up the remainder. In terms of clinical outcomes, excluding those with unknown outcomes, “Hospitalization - Initial or Prolonged” accounted for 20.3%, and death cases accounted for 17.8%, highlighting the potential risk of serious AEs related to Tofersen. In terms of temporal trends, the number of reports increased markedly in 2024 and 2025, reaching 160 cases (39.1%) and 208 cases (50.9%), respectively (Table 2).

**Table 2:** Basic information on AE reports for Tofersen.

Factor	Number of events (%)
Gender	
Female	176 (43.0)
Male	194 (47.4)
Unknown	39 (9.5)
Age	
18–64.9	176 (43.0)
65–85	68 (16.6)
>85	1 (0.2)
Unknown	164 (40.1)
Weight	
<50 kg	2 (0.5)
50-100 kg	34 (8.3)
>100 kg	3 (0.7)
Unknown	370 (90.5)
Reported countries	
United States	298 (72.9)
Italy	23 (5.7)
European Union	17 (4.2)
Japan	11 (2.7)
Spain	9 (2.2)
France	9 (2.2)
Australia	7 (1.7)
China	6 (1.5)
Switzerland	4 (1.0)
United Kingdom	4 (1.0)
Poland	4 (1.0)
Brazil	3 (0.7)
Canada	3 (0.7)
Germany	3 (0.7)
Israel	3 (0.7)
Belgium	1 (0.2)
Hrvatska	1 (0.2)
Hungary	1 (0.2)
Korea	1 (0.2)
Singapore	1 (0.2)
Reporter	
Consumer	194 (47.4)
Physician	120 (29.3)
Health professional	48 (11.7)
Pharmacist	19 (4.6)
Unknown	28 (6.8)
Induction time	
0–30 days	31 (28.2)
31–60 days	13 (11.8)
61–90 days	9 (8.2)
91–120 days	6 (5.5)
121–150 days	3 (2.7)
151–180 days	5 (4.5)
181–360 days	19 (17.3)
>360 days	24 (21.8)
Outcomes	
Hospitalization - Initial or Prolonged	83 (20.3)
Death	73 (17.8)
Other Serious (Important Medical Event)	40 (9.8)
Life-Threatening	6 (1.5)
Disability	2 (0.5)
Unknown	205 (50.1)
Reporter year	
2023	41 (10.0)
2024	160 (39.1)
2025	208 (50.9)

“European Union” indicates reports originating from the European region without specification of an individual country in FAERS.

The onset time indicated that 28.2% of cases experienced AEs within 30 days of treatment, with the incidence gradually decreasing thereafter. However, there was a slight increase in the sixth month, and 21.8% of patients still reported drug-related AEs after more than 12 months of treatment, indicating the need for attention to long-term drug safety. Notably, in the weighted signal proportion analysis, both the shape parameter and the upper limit of the 95% confidence interval were less than 1. This result suggests that the clinical priority signals for Tofersen exhibit an early failure pattern (Table 3).

**Table 3:** Time-to-onset analysis for signals with Tofersen.

Prioritization	Case	TTO (days)		Weibull distribution				Failure type
				Scale parameter		Shape parameter		
	n	Median (IQR)	Min–max	α	95% CI	β	95% CI	
Tofersen	110	97.5 (307.25)	1–2,317	179.61	135.21–238.59	0.69	0.60–0.80	Early failure

n, number of cases with available time-to-onset; TTO, time-to-onset; IQR, interquartile range.

### 3.2 Signal mining results

Results showed that Tofersen-related AEs involved 22 system organ classes (SOC). As shown in Table 4, the top four SOCs by report count were: Nervous system disorders (*n* = 209; ROR = 2.83; PRR = 2.46; EBGM = 2.46; IC = 1.3), Injury, poisoning, and procedural complications (*n* = 174; ROR = 1.68; PRR = 1.57; EBGM = 1.57; IC = 0.65), General disorders and administration site conditions (*n* = 129; ROR = 0.67; PRR = 0.71; EBGM = 0.71; IC = −0.49), and Musculoskeletal and connective tissue disorders (*n* = 113; ROR = 2.24; PRR = 2.11; EBGM = 2.11; IC = 1.08). This distribution pattern is generally consistent with the pharmacological characteristics and intrathecal administration route of Tofersen. Infections and infestations may reflect AEs related to injection procedures. In addition, we identified certain AE features concentrated in sensory systems, such as eye disorders and ear and labyrinth disorders, with relatively high signal strength, indicating the need for clinical vigilance. Respiratory, thoracic, and mediastinal disorders also showed signal strength; although there is no clear association with the known mechanism of action of Tofersen, they still warrant attention. Notably, ear and labyrinth disorders and respiratory, thoracic, and mediastinal disorders have not been documented in the current product labeling, and their potential causal relationships need to be further verified through large-scale clinical studies.

**Table 4:** Signal strength of AEs reports for Tofersen at the SOC level.

SOC	Case reports	ROR (95% CI)	PRR (χ2)	EBGM (EBGM05)	IC (IC025)
Nervous system disorders	209	2.83(2.43–3.3)	2.46(197.43)	2.46(2.11)	1.3(1.07)
Injury, poisoning and procedural complications	174	1.68(1.43–1.98)	1.57(40.01)	1.57(1.33)	0.65(0.41)
General disorders and administration site conditions	129	0.67(0.56–0.81)	0.71(18.32)	0.71(0.59)	-0.49(−0.76)
Musculoskeletal and connective tissue disorders	113	2.24(1.85–2.73)	2.11(69.36)	2.11(1.73)	1.08(0.77)
Investigations	91	1.56(1.26–1.94)	1.51(16.89)	1.51(1.22)	0.6(0.28)
Infections and infestations	73	1.38(1.08–1.75)	1.35(6.96)	1.35(1.06)	0.43(0.08)
Respiratory, thoracic and mediastinal disorders	63	1.33(1.03–1.72)	1.31(4.93)	1.31(1.02)	0.39(0.01)
Gastrointestinal disorders	36	0.39(0.28–0.54)	0.41(33.15)	0.41(0.3)	−1.28(−1.74)
Psychiatric disorders	34	0.6(0.43–0.85)	0.61(8.7)	0.61(0.44)	−0.7(−1.18)
Eye disorders	22	1.08(0.71–1.65)	1.08(0.13)	1.08(0.71)	0.11(−0.51)
Metabolism and nutrition disorders	16	0.73(0.44–1.19)	0.73(1.6)	0.73(0.45)	−0.45(−1.13)
Vascular disorders	13	0.61(0.35–1.05)	0.62(3.2)	0.62(0.36)	−0.7(−1.44)
Skin and subcutaneous tissue disorders	12	0.2(0.11–0.35)	0.21(38.08)	0.21(0.12)	−2.26(−2.97)
Cardiac disorders	10	0.4(0.21–0.74)	0.41(8.93)	0.41(0.22)	−1.3(−2.1)
Ear and labyrinth disorders	10	2.23(1.2–4.15)	2.22(6.7)	2.22(1.19)	1.15(0.12)
Renal and urinary disorders	7	0.35(0.17–0.73)	0.35(8.47)	0.35(0.17)	−1.5(−2.41)
Hepatobiliary disorders	3	0.33(0.11–1.04)	0.34(3.99)	0.34(0.11)	−1.58(−2.76)
Neoplasms benign, malignant and unspecified (incl cysts and polyps)	3	0.12(0.04–0.37)	0.12(19.39)	0.12(0.04)	−3.03(−4.12)
Immune system disorders	2	0.17(0.04–0.67)	0.17(8.35)	0.17(0.04)	−2.58(−3.78)
Product issues	1	0.06(0.01–0.4)	0.06(15.72)	0.06(0.01)	−4.12(−5.24)
Reproductive system and breast disorders	1	0.11(0.02–0.78)	0.11(7.27)	0.11(0.02)	−3.19(−4.38)
Blood and lymphatic system disorders	1	0.06(0.01–0.42)	0.06(15.05)	0.06(0.01)	−4.07(−5.19)

Among the 369 Tofersen-related AEs, 43 signals met the selection criteria across the four algorithms. Of these, the highest proportion was from the category of injury, poisoning, and procedural complications (9/43). EBGM-based signal ranking showed the strongest signals for neurological procedural complication (*n* = 9; ROR = 4506.99; PRR = 4467.35; EBGM = 4223.73; IC = 12.04), cerebrospinal fluid (CSF) red blood cell count positive (*n* = 4; ROR = 3455.02; PRR = 3441.51; EBGM = 3295.11; IC = 11.69), CSF white blood cell count increased (*n* = 8; ROR = 2987.34; PRR = 2963.98; EBGM = 2854.75; IC = 11.48), and CSF cell count increased (*n* = 5; ROR = 2646.74; PRR = 2633.81; EBGM = 2547.21; IC = 11.31). After excluding indication-related events, the most frequently reported AEs were procedural pain (*n* = 43; ROR = 96.83; PRR = 92.8; EBGM = 92.69; IC = 6.53), headache (*n* = 42; ROR = 4.07; PRR = 3.94; EBGM = 3.94; IC = 1.98), and post lumbar puncture syndrome (*n* = 34; ROR = 1977.65; PRR = 1911.95; EBGM = 1865.9; IC = 10.87). Additionally, other AEs related to injury, poisoning, and procedural complications included procedural headache, procedural dizziness, procedural vomiting, and procedural nausea, all of which should be closely monitored during clinical application. It is worth noting that certain central nervous system reactions (intracranial pressure increased, peroneal nerve palsy, papilloedema, and facial paralysis) and infections (radiculopathy, myelitis, pneumonia aspiration, and meningitis) were also observed. Furthermore, this study identified AEs related to CSF cytological and biochemical abnormalities, including CSF red blood cell count positive, CSF white blood cell count increased, CSF cell count increased, CSF protein increased, CSF test abnormal, and pleocytosis, which warrant thorough attention. After excluding symptoms related to disease progression and non-drug-related PTs, this study also identified pulmonary embolism as a serious AE, which requires close monitoring of specific patients in clinical practice (Table 5).

**Table 5:** The top 43 signal strength of AEs reports for Tofersen at the PT level.

SOC	PT	Case reports	ROR (95% CI)	PRR (χ2)	EBGM (EBGM05)	IC (IC025)
Injury, poisoning and procedural complications	Neurological procedural complication	9	4506.99(2295.39–8849.48)	4467.35(37996.14)	4223.73(2151.12)	12.04(2.37)
Investigations	CSF red blood cell count positive	4	3455.02(1266.69–9423.9)	3441.51(13172.62)	3295.11(1208.06)	11.69(0.99)
Investigations	CSF white blood cell count increased	8	2987.34(1470.5–6068.79)	2963.98(22822.34)	2854.75(1405.24)	11.48(2.18)
Investigations	CSF cell count increased	5	2646.74(1083.17–6467.34)	2633.81(12726.22)	2547.21(1042.44)	11.31(1.38)
Injury, poisoning and procedural complications	Post lumbar puncture syndrome	34	1977.65(1399.32–2794.99)	1911.95(63374.72)	1865.9(1320.25)	10.87(4.6)
Investigations	CSF protein increased	19	1946.85(1229.81–3081.96)	1910.71(35392.54)	1864.72(1177.93)	10.86(3.65)
Nervous system disorders	Amyotrophic lateral sclerosis^a^	43	1438.6(1057.27–1957.46)	1378.17(58142.44)	1354.09(995.16)	10.4(4.97)
Injury, poisoning and procedural complications	Procedural headache	6	919.97(410.39–2062.26)	914.58(5411.58)	903.91(403.23)	9.82(1.7)
Injury, poisoning and procedural complications	Procedural dizziness	4	765.89(285.52–2054.45)	762.9(3013.91)	755.46(281.63)	9.56(1.01)
Investigations	CSF test abnormal	3	462.27(148.34–1440.54)	460.92(1368.62)	458.2(147.03)	8.84(0.54)
Infections and infestations	Myelitis	8	328.08(163.39–658.79)	325.52(2577.44)	324.17(161.44)	8.34(2.17)
Nervous system disorders	Pleocytosis	3	323.59(103.94–1007.36)	322.64(957.95)	321.31(103.21)	8.33(0.54)
Injury, poisoning and procedural complications	Procedural vomiting	3	279.69(89.87–870.43)	278.87(827.66)	277.88(89.29)	8.12(0.54)
Injury, poisoning and procedural complications	Procedural nausea	3	232.98(74.89–724.82)	232.3(688.86)	231.61(74.45)	7.86(0.53)
Investigations	Light chain analysis increased	4	225.65(84.41–603.25)	224.77(888.54)	224.12(83.84)	7.81(1)
Nervous system disorders	Radiculopathy	7	128.8(61.21–271.04)	127.93(880.16)	127.72(60.7)	7(1.9)
Infections and infestations	Meningitis aseptic	9	126.11(65.39–243.2)	125.01(1105.42)	124.81(64.72)	6.96(2.3)
Injury, poisoning and procedural complications	Procedural pain	43	96.83(71.33–131.43)	92.8(3901.85)	92.69(68.28)	6.53(4.46)
Eye disorders	Papilloedema	6	86.08(38.56–192.14)	85.58(501.02)	85.48(38.3)	6.42(1.61)
Injury, poisoning and procedural complications	Procedural complication	6	58.57(26.24–130.73)	58.24(337.3)	58.19(26.07)	5.86(1.57)
Nervous system disorders	Intracranial pressure increased	4	45.03(16.86–120.24)	44.86(171.43)	44.83(16.79)	5.49(0.9)
Nervous system disorders	Peroneal nerve palsy	3	32.03(10.31–99.49)	31.94(89.87)	31.92(10.28)	5(0.42)
Infections and infestations	Meningitis	3	28.28(9.1–87.84)	28.2(78.67)	28.19(9.07)	4.82(0.41)
Injury, poisoning and procedural complications	Post procedural complication	7	26.57(12.64–55.89)	26.4(171.05)	26.39(12.55)	4.72(1.64)
Gastrointestinal disorders	Salivary hypersecretion	3	18.69(6.02–58.05)	18.64(50.07)	18.63(6)	4.22(0.34)
Nervous system disorders	Motor dysfunction^a^	3	18.05(5.81–56.08)	18(48.17)	18(5.79)	4.17(0.33)
Investigations	Sars-cov-2 test positive	3	16.71(5.38–51.91)	16.66(44.17)	16.66(5.36)	4.06(0.32)
Nervous system disorders	Facial paralysis	3	13.08(4.21–40.64)	13.05(33.38)	13.05(4.2)	3.71(0.26)
Respiratory, thoracic and mediastinal disorders	Respiratory failure^a^	14	12.09(7.14–20.49)	11.94(140.49)	11.94(7.05)	3.58(2.04)
Infections and infestations	Pneumonia aspiration	4	9.8(3.67–26.18)	9.77(31.5)	9.77(3.66)	3.29(0.53)
Respiratory, thoracic and mediastinal disorders	Acute respiratory failure^a^	3	9.57(3.08–29.72)	9.54(22.95)	9.54(3.07)	3.25(0.16)
Psychiatric disorders	Poor quality sleep	3	8.25(2.66–25.62)	8.23(19.05)	8.23(2.65)	3.04(0.11)
Respiratory, thoracic and mediastinal disorders	Respiratory disorder	4	8.22(3.08–21.96)	8.2(25.28)	8.2(3.07)	3.03(0.45)
Musculoskeletal and connective tissue disorders	Muscular weakness^a^	14	7.41(4.38–12.57)	7.33(76.62)	7.33(4.32)	2.87(1.61)
Respiratory, thoracic and mediastinal disorders	Respiratory distress	3	6.84(2.2–21.24)	6.82(14.91)	6.82(2.2)	2.77(0.03)
General disorders and administration site conditions	Inflammation	5	6.16(2.56–14.84)	6.14(21.52)	6.14(2.55)	2.62(0.54)
Musculoskeletal and connective tissue disorders	Musculoskeletal stiffness	8	5.41(2.7–10.85)	5.38(28.54)	5.38(2.68)	2.43(0.89)
Musculoskeletal and connective tissue disorders	Back pain	20	5.16(3.31–8.03)	5.08(65.69)	5.07(3.26)	2.34(1.45)
Musculoskeletal and connective tissue disorders	Mobility decreased^a^	6	4.86(2.18–10.85)	4.84(18.29)	4.84(2.17)	2.27(0.55)
Respiratory, thoracic and mediastinal disorders	Pulmonary embolism	7	4.76(2.26–10.01)	4.73(20.65)	4.73(2.25)	2.24(0.67)
Vascular disorders	Thrombosis	6	4.71(2.11–10.51)	4.69(17.43)	4.69(2.1)	2.23(0.52)
Gastrointestinal disorders	Dysphagia^a^	7	4.34(2.06–9.13)	4.32(17.87)	4.32(2.05)	2.11(0.58)
Nervous system disorders	Headache	42	4.07(2.99–5.54)	3.94(93.16)	3.94(2.89)	1.98(1.43)

a Indication-related PTs.

## 4 Discussion

With the acceleration of population aging, the disease burden caused by ALS is increasing (16). According to research models based on published country-specific ALS incidence estimates, the global number of ALS cases could increase from 222,801 in 2015 to 376,674 in 2040, a rise of 69%, with a larger proportion of the increase occurring in developing countries (17). Moreover, current treatment options remain limited and are unable to achieve effective disease control. FDA-approved ALS treatments such as riluzole, edaravone, and Relyvrio can delay disease progression to some extent but have limited overall efficacy, and some patients experience poor tolerance (18, 19). As the first antisense oligonucleotide (ASO) therapy targeting SOD1 mutation ALS, Tofersen shows unique advantages in reducing SOD1 protein levels and slowing neuronal damage (6). However, since its approval, clinical experience with Tofersen remains limited, and long-term safety data are still lacking. Furthermore, while Tofersen offers the advantage of innovative therapy, its high price has raised concerns about cost-effectiveness, further emphasizing the need for long-term safety monitoring. Based on pharmacovigilance research using the FAERS database, this study systematically analyzes the real-world AE data of Tofersen, aiming to identify potential safety signal patterns.

The results showed that the proportion of AE reports in males was higher than in females, which is consistent with the higher ALS incidence in men observed in epidemiological studies (20). The gender differences in AE reports may be multifaceted, potentially related to medication preferences and the neuroprotective effects of estrogen on motor neurons (21). However, in the context of spontaneous reporting systems, this finding may also be influenced by differential reporting behavior and variations in healthcare utilization. Further exploratory studies are needed to investigate the specific gender differences and their underlying factors (22). Notably, the geographical distribution of the data exhibited a marked North America-centric pattern, which is likely related not only to the inherent structure of the FAERS database but also to regional differences in pharmacovigilance systems, reporting intensity, drug accessibility, and post-marketing surveillance practices. This also reflects the limited representation of racial and ethnic diversity in the current evidence. In the future, more AE reports from different regions should be incorporated to improve the global generalizability of the data. Particularly after the approval of Tofersen by the NMPA in China (10), strengthened real-world safety monitoring in the Chinese population is warranted.

Regarding the composition of report sources, the proportion of spontaneous reports from consumers was roughly comparable to that from healthcare providers. This reflects both the active participation of patients in pharmacovigilance systems and the pivotal role of clinicians in drug safety monitoring, together forming a bidirectional flow of safety information. However, these sources may differ in reporting quality, clinical validation, and data completeness. Consumer reports are more likely to reflect subjective symptom perception, whereas healthcare professional reports tend to be more clinically structured (23).

Temporal analysis indicated that since the market launch of Tofersen, the volume of AE reports has shown a continuous upward trend. This trend is likely multifactorial, including increased drug exposure with expanded clinical use, heightened awareness among healthcare providers, and regulatory reporting requirements. Although the Weber effect suggests that AE reporting peaks within 2 years after drug approval (24), this does not necessarily indicate a subsequent short-term decline, as Tofersen is still being gradually introduced into clinical practice across different countries and regions worldwide. Therefore, continuous epidemiological surveillance and post-marketing safety studies are essential.

The onset time showed a U-shaped distribution. This temporal pattern may be attributed to three main reasons: intrathecal administration of Tofersen may induce early local tissue irritation; delivery via cerebrospinal fluid may lead to delayed central nervous system–related reactions; and the immunogenic potential of antisense oligonucleotide therapies may become more evident with prolonged exposure (25, 26). These characteristics underscore the importance of continuous AE monitoring. Regarding clinical outcomes, severe AEs requiring hospitalization (including new admissions or prolonged hospital stays) accounted for the highest proportion, highlighting the potential risk of severe complications with Tofersen. However, this finding should be interpreted in the context of spontaneous reporting systems, where serious outcomes are more likely to be reported, potentially leading to overrepresentation of severity. Notably, a considerable number of cases had unknown clinical outcomes, revealing gaps in follow-up completeness within the current pharmacovigilance system. Future studies should integrate multicenter long-term follow-up data to refine risk–benefit assessment frameworks.

As a drug acting on the central nervous system via the intrathecal route, Tofersen-related AEs are primarily categorized under injury, poisoning and procedural complications, nervous system disorders, and musculoskeletal and connective tissue disorders. This distribution is closely associated with its unique administration route and molecular mechanism of action.

### 4.1 Known adverse events

Our findings reveal disproportionality estimates consistent with clinical practice, particularly for neurological procedural complications, post-lumbar puncture syndrome, procedural headache, procedural dizziness, procedural vomiting, procedural nausea, procedural pain, procedural complications, and post-procedural complications. Some of these AEs exhibit notably high signal intensity. Studies suggest that signal strength in disproportionality analyses of marketed drugs is broadly consistent with relative risk estimates and may serve as a rough indicator of clinical relevance (27). These AEs may be attributed to three key factors: mechanical injury associated with lumbar puncture during intrathecal administration (28), chemical irritation related to the thiophosphonate backbone of Tofersen (29, 30), and alterations in cerebrospinal fluid dynamics potentially leading to autonomic dysfunction (31). Furthermore, symptom-burden AEs such as back pain and headache may negatively impact patient adherence (32).

Although consistent with previous findings on Nusinersen that intrathecal ASOs are associated with AEs such as post-lumbar puncture syndrome (33), interpretation of these signals should be made with caution. Given that the FAERS database predominantly includes orally and intravenously administered drugs, the disproportionality of certain signals related to intrathecal agents may be overestimated. That is, procedure-related AEs associated with intrathecal administration of Tofersen may be overestimated in FAERS due to differences compared with systemically administered therapies. Despite this limitation, the identified signals remain clinically meaningful, as they reflect real-world complications associated with lumbar puncture–based drug delivery, which is an essential component of Tofersen treatment. Clinicians should consider these AEs as important post-treatment monitoring indicators. To mitigate these risks, enhanced post-administration neurological assessments and proactive pain management strategies should be incorporated into routine clinical practice to facilitate early detection and management of potential complications.

It is noteworthy that certain central nervous system reactions and infections highlight potential risks, including radiculopathy, myelitis, and aseptic meningitis, which are consistent with adverse neurological events observed in clinical trials (8). Radiculopathy is characterized by radiating pain and sensory-motor abnormalities (34), and its mechanism may be secondary to mechanical injury caused by the puncture procedure or drug-induced inflammatory lesions around the nerve roots. In addition to mechanical injury, Tofersen, through inhibition of SOD1 protein, may induce local inflammatory or immune responses (35), leading to inflammatory lesions around the nerve roots. Moreover, drug-induced immune responses may also contribute to myelitis and other central nervous system inflammatory reactions (36), while puncture procedures may further increase the risk of infections (37), including aseptic meningitis (38) and other immune-mediated lesions. These findings emphasize the importance of enhanced individualized monitoring during Tofersen treatment, especially in the early stages and among high-risk patient populations.

### 4.2 Newly discovered risks

In this study, CSF red blood cell count positive exhibited the highest signal strength, with related findings including CSF white blood cell count increased and CSF protein increased. Tofersen is administered via intrathecal injection, which requires penetration of the dura mater and arachnoid membrane; during this procedure, damage to the intrathecal venous plexus or small blood vessels may occur. Additionally, ASOs can activate local immune responses, such as the release of pro-inflammatory mediators by microglial cells, leading to disruption of the blood–brain barrier and leakage of blood components into the cerebrospinal fluid, resulting in increased CSF red and white blood cell counts (39). The increase in CSF protein may be explained by several mechanisms. First, intrathecal administration may cause minor disruption of meningeal or small vascular structures during lumbar puncture, leading to contamination of CSF with plasma proteins and a consequent increase in measured CSF protein levels. Second, as an antisense oligonucleotide, Tofersen may induce local innate immune activation, including microglial and astrocytic responses, contributing to local inflammation and protein exudation (40).

Importantly, increased intracranial pressure and papilloedema were also found to be associated with Tofersen. Although the number of AE reports is limited, the signal strength is high. While no definitive studies have been conducted, these AEs may be explained by several potential mechanisms. First, Tofersen is administered via intrathecal injection to deliver the drug to the central nervous system; administration of a fixed volume (approximately 10 mL) into the intrathecal space may transiently influence cerebrospinal fluid dynamics and be associated with short-term changes in intracranial pressure due to a volume-related effect (41). Second, accumulation of ASOs in the subarachnoid space may reduce cerebrospinal fluid absorption (42). Furthermore, the thiophosphate backbone of the drug may activate microglial cells via the TLR3/8 pathway, leading to the release of pro-inflammatory cytokines such as IL-1β and TNF-*α*, which may contribute to vasogenic cerebral edema (39). Increased intracranial pressure may, in turn, result in secondary papilloedema. Animal studies suggest that this process is associated with glial activation mediated by TLR signaling, including increased IL-6 secretion by astrocytes (39). These findings indicate that clinicians should exercise caution when using Tofersen in patients with pre-existing intracranial conditions (e.g., brain tumors or cerebrovascular diseases).

Notably, through signal detection, this study also identified severe AEs such as pulmonary embolism. Pulmonary embolism is the third most common acute cardiovascular disease associated with death and disability (43). At present, the relationship between Tofersen and thromboembolic events remains uncertain. Several hypotheses may be considered. Cerebrospinal fluid contains various biologically active and potentially procoagulant components, including tissue factor and other coagulation-related proteins (44), and experimental evidence suggests that certain CSF constituents may participate in coagulation pathways under pathological conditions (45). In addition, mechanical stimulation associated with intrathecal injection may induce neuroinflammatory responses mediated by proinflammatory cytokines, which can disrupt blood–brain barrier integrity and increase its permeability (46). The resulting exposure or redistribution of coagulation-related factors may theoretically contribute to a prothrombotic microenvironment. Moreover, ASOs may have immunogenic potential (35), and immune activation has been implicated in thrombosis (47). Procedure-related factors, including infection or inflammatory complications associated with lumbar puncture, may also contribute to activation of coagulation pathways, as reported in other clinical settings (48). However, these mechanisms remain speculative in the context of Tofersen and have not been specifically demonstrated. Therefore, although pulmonary embolism emerged as a signal in the FAERS database, this finding should be interpreted with considerable caution given the limited number of cases, potential reporting bias, and lack of mechanistic validation. Further post-marketing studies and clinical evidence are required to determine whether this represents a true drug-related risk or a procedure- and population-related confounding phenomenon.

This study has several limitations. First, the relatively small number of reports, particularly in certain subgroup with small case counts, represents an important constraint. Disproportionality metrics such as ROR may be unstable under these conditions, and extreme values can arise from sparse data or small-cell effects. Accordingly, inflated ROR values may reflect statistical artifacts rather than true disproportional reporting. Therefore, signals identified from a limited number of reports should be interpreted with caution and regarded as hypothesis-generating rather than confirmatory. Second, as FAERS is based on spontaneous reporting, it is subject to reporting bias and underreporting. In addition, this study relies on existing AE reports and lacks detailed information on patients characteristics, medication history, and treatment regimens, making it difficult to control for potential confounders. Third, the predominance of orally and intravenously administered drugs in FAERS may introduce comparator bias, potentially overestimating signals for intrathecal agents like Tofersen due to procedure-related AEs. Finally, geographic differences in the data, particularly the North American-centric distribution, limit representation of the global population and racial diversity. Future studies should expand geographic coverage and incorporate more clinical data with longitudinal follow-up to achieve a more comprehensive understanding of Tofersen’s safety profile.

## 5 Conclusion

This study is the first to systematically conduct a post-marketing pharmacovigilance analysis of Tofersen. It not only validated the common AEs recorded in the drug labeling but also identified novel risk signals with potential clinical significance. The study established a comprehensive safety profile encompassing procedure-related AEs, neurological reactions, and immune responses.

Particular attention should be given to AEs related to CSF cytology and biochemical abnormalities, such as CSF red blood cell count positive, CSF white blood cell count increased, and CSF protein increased, as well as AEs associated with injury, poisoning, and procedural complications, including post-lumbar puncture syndrome, procedural pain, and procedural headache. Central nervous system AEs, such as increased intracranial pressure, peroneal nerve palsy, papilloedema, and facial paralysis, also warrant attention. Although radiculopathy, myelitis, aspiration pneumonia, and meningitis have a relatively low overall occurrence, their high signal strength suggests potential clinical importance, necessitating further mechanistic studies. Notably, this study is the first to identify a thrombosis-related risk signal. While the exact mechanism linking pulmonary embolism to Tofersen remains unclear, these findings highlight potential risks in specific patient populations, particularly involving thrombosis formation, immune responses, or vascular injury.

Future research should integrate genomics and patient-specific risk factors to better predict potential AEs. Additionally, further causality studies are necessary to establish a direct link between these AEs and Tofersen.

## Data Availability

The original contributions presented in the study are included in the article/supplementary material, further inquiries can be directed to the corresponding authors.
